# Effects of Roux-en-Y gastric bypass on the metabolic profile and systemic inflammatory status of women with metabolic syndrome: randomized controlled clinical trial

**DOI:** 10.1186/s13098-023-00986-2

**Published:** 2023-02-14

**Authors:** Lucenda A. Felipe, André L. L. Bachi, Miriã C. Oliveira, Sandra M. B. P. Moreira, João Pedro R. Afonso, Maria E. M. Lino, Vitória Paixão, Carlos H. M. Silva, Rodolfo P. Vieira, Sergio Vencio, Elias I. Jirjos, Carlos A. Malheiros, Giuseppe Insalaco, Wilson R. Freitas Júnior, Luis V. F. Oliveira

**Affiliations:** 1 Post-Graduation Program in Health Sciences, Santa Casa of Sao Paulo Medical School, Sao Paulo, SP 01221-010 Brazil; 2 Post-Graduation Program in Health Sciences, Santo Amaro University (UNISA), São Paulo, SP Brazil; 3 Human Movement and Rehabilitation Post Graduation Program, Evangelical University of Goiás (UniEVANGELICA), Anápolis, GO Brazil; 4 Scientific Initiation Program, Evangelical University of Goiás, (UniEVANGELICA), Anápolis, GO Brazil; 5 Department of Otorhinolaryngology, ENT Lab, Federal University of São Paulo (UNIFESP), São Paulo, SP 04021-001 Brazil; 6 Institute of Pharmaceutical Sciences, Goiania, (GO) Brazil; 7 Institute for Biomedical Research and Innovation, National Research Council of Italy (CNR), 90146 Palermo, Italy

**Keywords:** Metabolic syndrome, Severe Obesity, Roux-en-Y Gastric Bypass, Inflammation, Metabolic Profile

## Abstract

**Background:**

Obesity remains a public health problem worldwide. The high prevalence of this condition in the population raises further concerns, considering that comorbidities are often associated with obesity. Among the comorbidities closely associated with obesity, metabolic syndrome (MS) is particularly important, which potentially increases the risk of manifestation of other disorders, such as the prothrombotic and systemic pro-inflammatory states.

**Methods:**

A randomized, controlled clinical trial was performed involving female patients (n = 32) aged between 18 and 65 years, with a clinical diagnosis of MS, with severe obesity undergoing Roux-en-Y gastric bypass (RYGB). The study design followed the Consolidated Standards of Reporting Trials statement (CONSORT). Lipid profile, blood glucose and adipokines (adiponectin, leptin, and resistin) and (cytokines IL-1β, IL-6, IL-17, IL-23, and TNF-α) in blood plasma samples were evaluated before and six months after RYGB.

**Results:**

Patients undergoing RYGB (BSG) showed a significant improvement from preoperative grade III obesity to postoperative grade I obesity. The results showed that while HDL levels increased, the other parameters showed a significant reduction in their postoperative values when compared not only to the values observed before surgery in the BSG group, but also to the values obtained in the control group (CG). As for systemic inflammatory markers adiponectin, leptin, resistin, IL-1β, IL-6, IL-17, IL-23 and TNF- α it was observed that the levels of resistin and IL-17 in the second evaluation increased significantly when compared to the levels observed in the first evaluation in the CG. In the BSG group, while the levels of adiponectin increased, the levels of the other markers showed significant reductions in the postoperative period, in relation to the respective preoperative levels. The analysis of Spearman’s correlation coefficient showed a significant positive correlation between IL-17 and IL-23 in the preoperative period, significant positive correlations between TNF-α and IL-6, TNF-α and IL-17, IL-6 and IL-17, and IL-17 and IL-23 were observed postoperatively.

**Conclusions:**

According to our results, the reduction of anthropometric measurements induced by RYGB, significantly improves not only the plasma biochemical parameters (lipid profile and glycemia), but also the systemic inflammatory status of severely obese patients with MS.

*Trials registration* NCT02409160

## Background

Obesity remains a public health problem worldwide. Its prevalence has been increasing globally in recent decades, even in developing countries. According to the World Health Organization (WHO), in 2016, the global prevalence of overweight adults over the age of 18 years was approximately 1.9 billion, of which 38% were men and 40% were women, and 650 million were obese (13%). The high prevalence of this condition in the population raises further concerns, considering that comorbidities are often associated with obesity [1, 2].

Among the comorbidities closely associated with obesity, metabolic syndrome (MS) is particularly important, which potentially increases the risk of manifestation of other disorders, such as the prothrombotic and systemic pro-inflammatory states [3, 4]. In addition, the prolonged high glucose levels that are associated with MS, induce a prolonged stimulation of the pancreas to release insulins, causing insulin resistance and type 2 diabetes mellitus (DM2). Moreover, increases in the arterial blood pressure, circulating triglycerides (TG), and interleukin-6 (IL-6)—an inflammatory cytokine—have been observed, which may contribute to a pro-inflammatory state and increase the risk of cardiovascular disease [5].

It is widely accepted that both a chronic low-grade inflammation and the immune system are important factors in obesity. A significant infiltration of leukocytes, particularly macrophages, can be observed in the tissues with altered profiles, leading to a pro-inflammatory state and consequent increase in systemic inflammation [6]. In addition to the increased levels of inflammatory cytokines, such as IL-6 and tumor necrosis factor-alpha (TNF-α), the production or release of other adipose tissues, especially visceral, leads to the production of or release from other tissues by the adipocyte itself, commonly known as adipokines, which contribute to the development of insulin resistance and atherosclerosis. Obesity imposes a particular condition and the release of adiponectin, a molecule with pro-inflammatory properties, in contrast to the increased production and release of leptin and resistin, which involves the production and two reactions, by the lesser role of adipose tissue, improving and accentuating the manifestation of low-grade chronic inflammation associated with obesity and MS [7].

Consequently, certain non-invasive approaches, such as changes in pharmacological treatment, lifestyle, dietary re-education, combinations, and modifications, are used to minimize, or even reverse obesity and MS [8, 9]. With the evolution of surgical techniques and procedures, bariatric surgery has proven to be an effective and safe approach to improve the patient's overall clinical condition. Bariatric surgery is currently the most widely used therapeutic option for severe MS and among MS patients, the Roux-en-Y gastric bypass (RYGB) procedure is particularly important; a surgical procedure that is characterized by a restriction and lower absorption, owing to a small gastric pouch and a total bypass of the duodenum and proximal jejunum [10–12]. It is worth noting that reducing body weight using bariatric surgery is effective in decreasing the severity of chronic inflammation, and therefore, the development of comorbidities associated with obesity [13].

Therefore, to understand the benefit of bariatric surgery by RYGB in women with severe obesity and MS, whose inflammatory profile (adiponectin, resistin, leptin, TNF-α, IL-1β, IL-6, IL-17, and IL-23) is available, an improvement in the understanding and description of the changes in the group of parameters before and after the surgery in this population is extremely important for clinical practice. The main objective of this study was to verify the effects of weight loss induced by RYGB on the circulating levels of systemic inflammatory markers adiponectin, resistin, leptin, TNF-alpha, IL1-beta interleukins, IL-6, IL-8, IL-17, IL-23 and IGF-1 in women with severe obesity and MS.

## Methods

### Study participants

We conducted a randomized, controlled, clinical trial at the Faculty of Medical Sciences of Santa Casa de São Paulo. We recruited female patients (n = 32) aged between 18 and 65 years, with a clinical diagnosis of MS and severe obesity (Body mass index (BMI) ≥ 40 kg/m^2^ or 35–39.9 kg/m^2^ associated with comorbidities) who were eligible for bariatric surgery. The exclusion criteria were clinical and/or mental instability, BMI > 55 kg/m^2^, unrealistic postoperative target weight and/or unrealistic expectations regarding surgical treatment, active cancer, pregnancy, lactation, or planning pregnancy within 2 years, and a diagnosis of psychiatric disorders (schizophrenia or depression).

### Recruitment, randomization and sample size

**Fig. 1:**
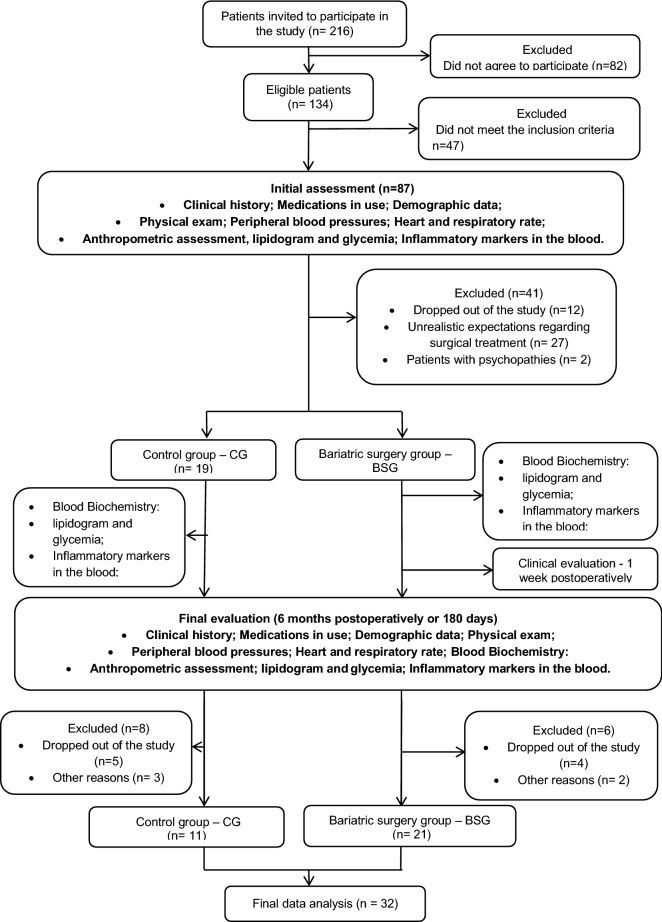
Study flowchart according to the CONSORT statement

The recruitment was performed at the obesity surgery outpatient clinic of Santa Casa de Misericórdia de São Paulo from January to December 2019. All the recruited volunteers signed a free and informed consent form that was previously approved by the National Ethics Committee in Research with Human Beings of Santa Casa de Misericórdia (Process number 178/2012) and registered in ClinicalTrials.gov NCT02409160. The study was conducted in accordance with the Regulatory Norms for Research Involving Human Subjects of the National Health Council of the Ministry of Health, RESOLUTION No. 466, on December 12, 2012 [14]. The study design followed the CONSORT (CONsolidated Standards of Reporting Trials) [15], as shown in Fig. 1.

The sample size was determined using G-Power software (version 3.1.2—Universitat Kiel, Germany), with an α error of 0.05 and power of 1−β error = 0.95. Based on the study of Freitas Jr. et al. [16], who studied plasma TNF-α levels in severely obese patients before and after bariatric surgery, an arbitrary moderate effect size (Cohen's d) of 0.8 was assumed, and with the calculation using a difference between two dependent means (matched pairs) t-test, the total sample size was 19 for BSG. In addition, since the CG did not show any differences in the same study assessed and that the main endpoint was evaluating the effect of bariatric surgery, we assumed that a group of 11 volunteers could be useful to perform this study [16].

All patients involved in this study were randomly allocated to the surgery and control groups. Because they were patients of the same gender and were composed of only two groups, the simple randomization procedure was adopted through a numerical sequence generated by computer in the proportion of 2:1. The consecutively generated numbers that indicated the assignment for each group were placed in sealed envelopes, later opened by the patient and stored by a research assistant. This entire process was carried out by a research assistant, avoiding the participation of researchers involved in the study. The statistician was also unaware of the composition of the data sheet.

### Clinical assessment

All volunteers were initially evaluated to obtain data regarding medical and surgical history, concomitant medications, demographic data, and anthropometric measurements. Blood samples were collected for biochemical analyses and inflammatory markers. All patients involved in the study had a confirmed clinical diagnosis of metabolic syndrome due to severe obesity. Thereafter, the volunteers who did not undergo bariatric surgery were a part of the control group (CG, n = 11), and those who underwent RYGB were allocated to the bariatric surgery group (BSG, n = 21). The CG volunteers were instructed to maintain their routine, especially regarding physical activities and eating habits, and to inform the researchers of any changes in their routine. A second evaluation was performed, and blood samples were collected 6 months after the first evaluation in the CG or 6 months after the bariatric surgery in the BSG group. All assessments were performed by the same team of researchers, physicians, and physical therapists.

### Bariatric surgery

All procedures related to bariatric surgery followed the previous description presented by Freitas et al. [16]. Briefly, all patients were operated on by three surgeons who alternated between being the surgeon and two assistants in each surgery. After general anesthesia, the patients were placed in the supine position with a sequential compression device for prophylaxis against deep vein thrombosis. The abdominal incision was marked, starting 2 cm below the xiphoid process and extending to 7 cm above the umbilicus. The surgery was performed by RYGB reconstruction, with a small Capella-type bag with gastrointestinal anastomosis in two sutures, one with continuous 4–0 Vicryl and the other with 3–0 seromuscular cotton sutures, with lateral anastomosis (1.5 cm in diameter). Silastic rings were not placed. The alimentary loop was 100 cm long, and the biliopancreatic loop was 70 cm long with 3–0 Vicryl, with lateral enteroanastomosis (4 cm in diameter) with continuous sutures in two layers.

### Blood sampling

Venous blood samples (5 mL) were collected after 12 h of fasting, through cubital venipuncture and collected in vacuum tubes (Vacuette do Brasil Ltda, Campinas, Brazil) with ethylenediaminetetraacetic acid anticoagulant (EDTA K3) to obtain plasma after centrifugation for 10 min at 2000 rpm. The samples were collected at baseline and 6 months after the first evaluation in the CG and 6 months after the surgery in the BSG group.

### Determination of lipid profile and glycemia

Plasma concentrations of total cholesterol (TC), high-density lipoprotein (HDL), low-density lipoprotein (LDL), TG, and glucose were determined using commercial kits (Gold Análise Diagnostica Ltda, MG, Brazil) and the SpectraMax i3 system (Molecular Devices, Sunnyvale, CA, USA).

### Determination of inflammatory parameters

Plasma concentrations of adipokines (adiponectin, leptin, and resistin) and cytokines (IL-1β, IL-6, IL-17, IL-23, and TNF-α) were determined using commercial enzyme-linked immune-assay kits (R&D Systems, Minneapolis, MN, USA), BioLegend (Sellex Inc., Washington, DC, USA), and Invitrogen (Thermo Fisher Scientific, Massachusetts, MA USA), according to the manufacturer's recommendations. The concentration of each parameter was calculated using an appropriate standard curve (according to the manufacturer’s instructions). The correlation coefficients of all standard curves ranged from 0.95 to 0.99, while the intra-assay coefficients of variance ranged from 3 to 5%, and the inter-assay coefficients of variance ranged from 8 to 10%.

### Statistical analysis

Initially, the data obtained were evaluated for normality using the Shapiro–Wilk D'Agostino, and Pearson tests, and variance homogeneity was analyzed using the Levene’s test. The parameters that showed a normal distribution (parametric data) were presented as mean and standard deviation, and the difference between the means of these variables was evaluated using the Student’s t-test. One-way analysis of variance with Dunn’s post-test was used to compare the variables in the CG and BSG group. Pearson’s correlation test was used to analyze possible trends and product-moment correlations.

The parameters that deviated from the normal distribution (non-parametric data) were presented as median and interquartile range, and the difference between their values was evaluated using the Wilcoxon test. In addition, the Friedman test was used to compare the variables obtained in the CG and BSG group. The Spearman correlation test was used to analyze possible trends and product-moment correlations. Statistical analysis was performed using the Statistical Package for Social Sciences (SPSS 21.0^®^, Chicago, Illinois, USA). The significance level was set at 5% for all tests (p < 0.05).

## Results

Table 1 presents the anthropometric characteristics of the volunteers involved in this study, who were divided into two groups, CG (n = 11) and BSG (n = 21). During the development of this clinical trial, eight patients were lost to follow-up in the control group and six in the experimental group. This loss is justified in the control group since these patients obtained surgical procedures in other hospitals. Regarding the patients in the bariatric surgery group, six patients were lost to follow-up because they refused to return to the hospital for postoperative examinations. Despite the follow-up losses, the number of patients involved in the final analysis exceeded the sample size, not affecting data analysis. The mean age of the volunteers in both the groups was similar (42 years), and all of them weighed more than 110 kg with grade III obesity. When analyzing the impact of significant weight loss and BMI reduction in the volunteers from the BSG group after surgery and comparing them to the pre-surgery values, it was shown that the difference between the BMI values in the CG and BSG group was initially 0.75 ± 0.34 (first assessment), which increased to 13.73 ± 0.6 after surgery (second assessment). Based on these observations, the volunteers in the BSG group showed a significant improvement from grade III obesity preoperatively to grade I postoperatively.

**Table 1:** Anthropometric characteristics of the patients involved in the study

Control Group (n = 11)				Bariatric Surgery Group (n = 21)			*p*	
Variables	Avaliation 1	Avaliation 2	*p*	Pre-BS	Post-BS	*p*	Pre BS	Post BS
Age (years)	42.09 ± 12.69			42.62 ± 10.83				
Weight (kg)	112.64 ± 17.12	114.45 ± 16.84	ns	124.1 ± 18.56	87.20 ± 14.90	***	ns	***
BMI (kg/m^2^)	46.35 ± 5.46	47.10 ± 5.12	ns	46.3 ± 6.3	32.65 ± 5.7	***	ns	***

*BS* bariatric surgery, p: statistical significance value, *BMI* body mass index, *ns* not significant

^*^statistically significant difference between the compared values

**correspond to *p*<0.01

*** correspond to *p*<0.001

Table 2 shows the plasma levels of TC and its fractions (LDL, HDL, and TG), and blood glucose levels obtained in the first and second evaluations in the CG volunteers. These times corresponded to the pre- and post-bariatric surgery for the volunteers in the BSG group. The results showed that while HDL levels increased, the other parameters showed a significant reduction in their values in the postoperative period when compared not only to the values observed before the surgery in the BSG group, but also to the values obtained in the CG.

**Table 2:** Plasma biochemical values (mg/dL) of total cholesterol, low density lipoprotein, high density lipoprotein, triglycerides and blood glucose

Control Group (n = 11)			*p*	Bariatric Surgery Group (n = 21)		*P*	*p*	
Variables	Avaliation 1	Avaliation 2		Pre-BS	Post-BS		PreBS	PostBS
TC	195.36 ± 23.34	194.00 ± 21.52	ns	202.90 ± 34.57	125.62 ± 24.15	****	ns	****
LDL	116.00 ± 35.92	121.18 ± 23.97	ns	131.00 ± 27.15	95.14 ± 20.36	****	ns	**
HDL	41.45 ± 6.95	44.18 ± 6.72	ns	44.59 ± 15.74	53.30 ± 7.74	*	ns	**
TG	125.55 ± 42.22	124.82 ± 42.03	ns	164.57 ± 73.24	89.95 ± 25.33	****	ns	**
Blood glucose	92.82 ± 8.06	104.27 ± 6.86	ns	105.7 ± 14.36	85.76 ± 8.42	***	ns	***

*BS* bariatric surgery, *TG* triglycerides, *TC* total cholesterol, *LDL* low density lipoprotein, *HDL* high density lipoprotein, *ns*: not statistically significant

^*^statistically significant difference between the values found in the pre and post-bariatric surgery moments

^*^p < 0.05

^**^p < 0.01

^***^p < 0.001

^****^p < 0.0001

**Fig. 2:**
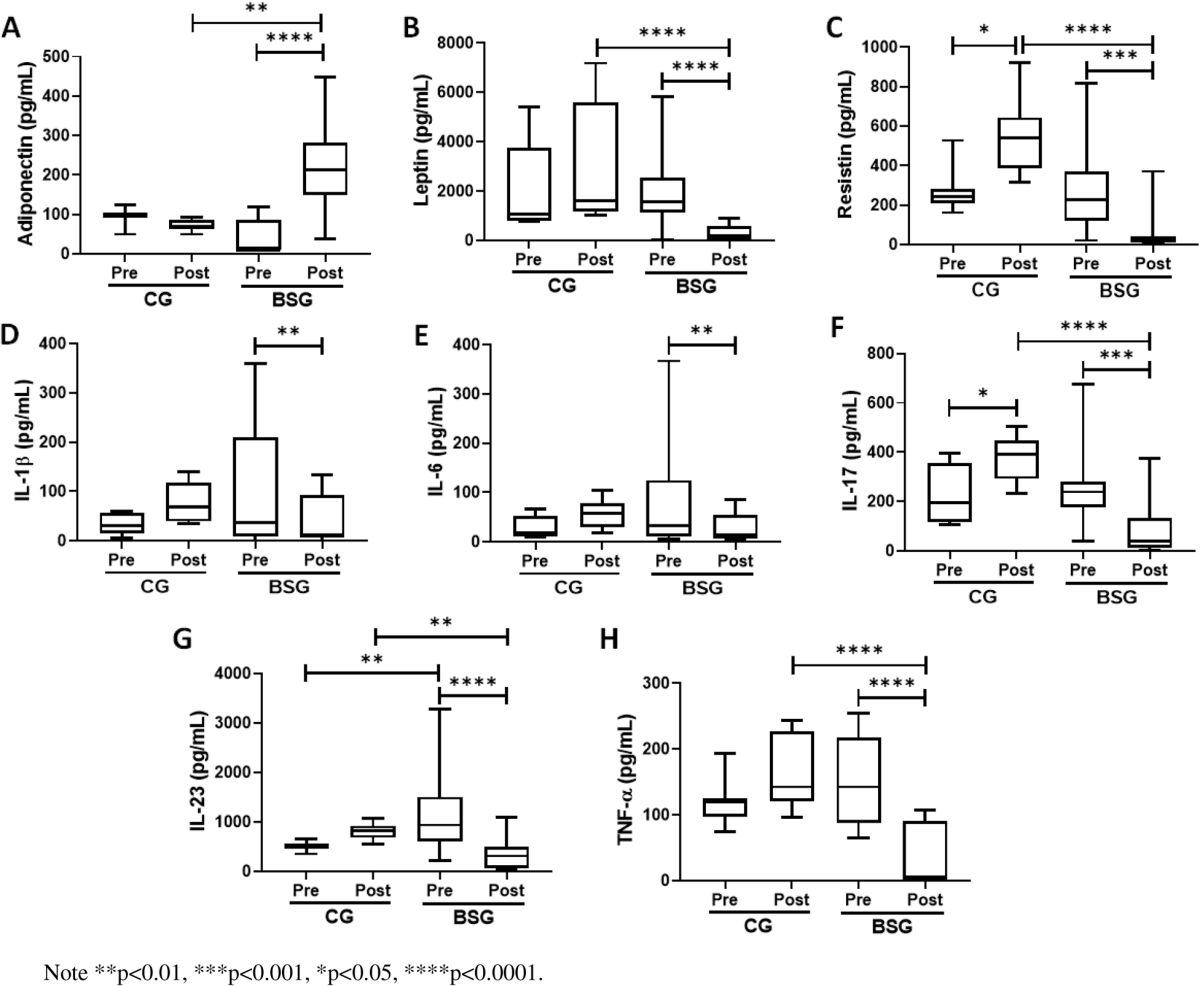
Plasma concentration of inflammatory markers (in pg/mL) adiponectin, leptin, resistin, IL-1, IL-6, IL-17, IL-23, TNF- of the patients involved in the studyNote.

Figure 2 shows the results of the evaluation of systemic inflammatory markers adiponectin (A), leptin (B), resistin (C), IL-1β (D), IL-6 (E), IL-17 (F), IL-23 (G), and TNF-α) in the volunteers who participated in the study in the two groups, CG and BSG. The results showed that the levels of resistin (B) and IL-17 (F) in the second evaluation significantly increased when compared to the levels observed in the first evaluation in the CG. In the BSG group, while adiponectin (A) levels reduced, the levels of the other markers showed statistically significant reductions postoperatively, compared to the respective preoperative levels. Furthermore, in the inter-group analysis, it was found that IL-23 (G) levels in the BSG group preoperatively were significantly higher than those in the CG. The levels of adiponectin (A) were higher, while the levels of leptin (B), resistin (C), IL-17 (F), IL-23 (G), and TNF-α (H) were lower in the BSG group postoperatively than in the CG.

Table 3 presents the results of the Spearman correlation coefficient analysis for the groups of volunteers participating in this study. In the CG, no significant correlation was found at any of the studied time-points. With regard to the BSG group, while a significant positive correlation between IL-17 and IL-23 was observed preoperatively, significant positive correlations between TNF-α and IL-6, TNF-α and IL-17, IL-6 and IL-17, and IL-17 and IL-23 were observed postoperatively.

**Table 3:** Spearman’s correlation coefficient of tumor necrosis factor-alpha cytokines and interleukins IL-1b, IL-6, IL-17 and IL-23 of the patients involved in the study

Correlation	Control group				Bariatric surgery group			
	Pre		Post		Pre		Post	
	*r-value*	*p-value*	*r-value*	*p-value*	*r-value*	*p-value*	*r-value*	*p-value*
TNF-α × IL-1β	0.2694	0.4188	0.5727	0.0708	− 0.1277	0.9866	0.3697	0.0990
TNF- α × IL-6	− 0.1689	0.6161	− 0.227	0.5033	− 0.0656	0.6704	0.4826	0.0266
TNF- α × IL-17	0.1138	0.7393	− 0.372	0.2607	0.3059	0.2893	0.5424	0.0111
TNF- α × IL-23	0.4246	0.1924	− 0.054	0.8812	0.2585	0.7728	0.2363	0.3023
IL-1β × IL-6	0.1438	0.6685	0.45454	0.1634	− 0.1648	0.9888	0.3778	0.8280
IL-1 β × IL-17	− 0.3508	0.2884	− 0.2363	0.4853	− 0.0584	0.8013	0.1891	0.4114
IL-1 β × IL-23	− 0.4566	0.1583	0.2	0.5574	− 0.0428	0.8536	0.1893	0.4109
IL-6 × IL-17	− 0.0045	0.9949	0.09090	0.7963	0.3645	0.1042	0.7277	0.0001
IL-6 × IL-23	− 0.3470	0.2925	0.13636	0.6937	0.0939	0.6853	0.3501	0.1196
IL-17 × IL-23	0.3690	0.2628	0.45454	0.1634	0.4339	0.0493	0.4413	0.0451

*TNF-a*  tumor necrosis factor-alpha, *IL-1b*  interleukins 1b, *IL-6* interleukins 6, *IL-17*  interleukins 17, and interleukins 23

## Discussion

Our results showed that patients with severe obesity and MS who underwent bariatric surgery using the RYGB technique had an improvement in all anthropometric, biochemical, and inflammatory parameters when compared to patients with the same conditions who did not undergo surgery. Interestingly, significant positive correlations were observed between the evaluated cytokines only after the bariatric surgery.

The accumulation of body fat is associated not only with aging, but also with the adoption of sedentary behaviors and the intake of hypercaloric foods which, over time, induce a natural reduction in metabolism and hormonal changes that can substantially contribute to the increase in the adiposity levels in the body [17].

The increase in body weight associated with the accumulation of abdominal fat in women may also be promoted by menopause. The cessation of ovarian function causes a reduction in metabolism, lean body mass, and energy expenditure in physical exercise, in addition to causing greater accumulation of fat in the adipose tissue. These conditions together contribute to a higher risk of obesity and the development of cardiovascular diseases in women [18].

Pontiroli and Morabito reported in a meta-analysis that patients undergoing bariatric surgery not only have reduced long-term mortality, but also a reduced risk of mortality from cardiovascular diseases [19] Corroborating this information, Yan et al. reiterated in their systematic review and meta-analysis that bariatric surgery using the RYGB technique promotes the reversal of DM2 in the short and medium- terms, improves the metabolic state, and reduces cardiovascular risk factors [20]. A study conducted on 47 obese women who underwent RYGB technique, demonstrated a considerable decrease in fasting blood glucose from 119.0 mg/dL to 94.6 mg/dL at 3 months and to 86.2 mg/dL at 12 months postoperatively [21]. This can be explained by the reduced length between the stomach and the intestine and increased production of incretins in anticipation of food in the terminal ileum, which regulates glucose metabolism by inhibiting insulin secretion [22]. Therefore, this corroborates with our findings of a significant reduction in circulating glucose levels in patients in the BSG group postoperatively.

Similarly, bariatric surgery with RYGB also showed a remarkable ability to reverse dyslipidemia and obesity. A systematic review and meta-analysis by Carswell et al. [23] reported that, in 7815 individuals undergoing RYGB, there was a significant reduction in plasma levels of TC, LDL, and TG (p < 0. 0.00001, p < 0.00001, and p < 0.00001, respectively) 1–3 months postoperatively. Conversely, plasma HDL levels increased after RYGB, especially after one year (p < 0.00001) [23]. Therefore, both the reduction in TC, LDL, and TG levels, and the increase in HDL levels observed in patients in the BSG group in our study corroborate with the data reported in this review.

As previously mentioned, RYGB promotes the reversal of dyslipidemia and obesity. Interestingly, obesity or excess fat in the adipose tissue, was identified as a metabolically active state in 2005; adipose tissue is considered a secretory organ and responsive to modulating signals such as appetite, energy balance, insulin sensitivity, reproduction, endocrine function, bone metabolism, immunity, and inflammation. [24].

The adipose tissue of obese individuals shows a marked infiltration by macrophages that promote the increased production and secretion of various pro-inflammatory mediators, thus characterizing the inflammation associated with obesity [25]. Thus, obesity is already established in the scientific literature as a low-grade inflammatory state called “Inflammome” characterized by chronic increases in the circulating levels of inflammatory markers [26]. A typical pattern of inflammation originates from the adipose tissue, which is consistent with the association between inflammatory markers and central adiposity [21, 22].

Among the various pro-inflammatory molecules associated with obesity and MS, leptin is particularly important, which is also a marker of long-term energy stores. Interestingly, both leptin and another molecule, resistin correlate with other inflammatory markers of obesity, independent of BMI [27]. Studies have also identified a close interrelationship between leptin and TNF-α, since the latter induces the production of leptin, which in turn, induces the production of TNF-α by macrophages infiltrating the adipose tissue [28, 29]. In contrast to the pro-inflammatory action of leptin, adiponectin has anti-inflammatory activities, although its interaction with TNF-α is still unclear [30].

It is widely accepted that adiponectin, which is produced and secreted preferentially by adipocytes, has remarkable anti-inflammatory and antiatherogenic properties, and is also capable of modulating glucose metabolism [31, 32]. In the context of RYGB surgery, the postoperative circulating levels of adiponectin increase in conjunction with the improved insulin sensitivity and reduced blood pressure [33–35]. Moreover, while adiponectin levels rise after RYGB, circulating leptin levels decrease [36].

Studies have shown significantly reduced levels of leptin in patients after RYGB surgery compared with the levels before surgery [37, 38] in individuals with normal weight [39], overweight [40], and obese individuals. [41–43]. Furthermore, lower leptin levels after RYGB correlate with significant reductions in body weight, body fat [44], and BMI [45, 46].

Therefore, these reports on adiponectin and leptin in patients undergoing RYGB surgery corroborate our results. Serum adiponectin levels significantly increased and leptin levels significantly reduced postoperatively when compared to the preoperative values and the values observed in the CG.

Resistin is another important molecule produced by the adipose tissue in inflammation [47, 48]. Studies have shown that, in humans, resistin has autocrine, paracrine, and endocrine actions, which affect a wide range of cell and tissue types [49, 50]. Further, the circulating level of resistin is positively associated with inflammatory biomarkers such as TNF-α, IL-1β, and IL-6, and diseases such as DM2 and atherosclerosis. [51] Studies have shown that circulating levels of resistin increase significantly with increasing BMI [52] and that obese patients have higher resistin levels than non-obese individuals [46].

Circulating levels of resistin are not generally altered after bariatric surgery [53]. However, a significant reduction in resistin levels was observed in patients with substantial weight loss [54]. For example, obese patients whose BMI reduced by approximately 50% after bariatric surgery showed significant improvement in resistin, leptin, and IL-6 levels, an increase in adiponectin, and increased insulin sensitivity [55]. Interestingly, our findings contribute to the data in the context of obesity and bariatric surgery, as they demonstrated that over time, resistin levels significantly increased in patients in the CG, which may indicate a worsening of their condition leading to adverse consequences. Conversely, patients who underwent RYGB surgery showed a significant reduction in circulating resistin levels, which may reflect a better inflammatory state in these patients. Corroborating this improvement in the inflammatory status in patients after RYGB surgery, together with the reduction in resistin levels, we also observed a decrease in the levels of pro-inflammatory molecules such as IL-1β, IL-6, IL-17, IL-23, and TNF-α.

In the context of obesity and MS, TNF-α is secreted by macrophages from the stromal vascular tissue associated with adipose tissue. TNF-α is a pro-inflammatory factor that plays a crucial role in the pathogenesis of insulin resistance. Persistently high levels of TNF-α cause phosphorylation of substrate-1 of the insulin receptor, preventing its pairing. Consequently, it prevents the signal generated by the binding of insulin to the surface receptor from activating the translocation of GLUT-4 to the membrane, especially in muscle and liver cells [56, 57].

Thus, the reduction in the circulating levels of TNF-α observed postoperatively in the BSG group after bariatric surgery with RYGB, when compared to their preoperative values and those in the CG, might have induced the normalization of blood glucose levels, thus favoring the reversal of DM2 by reducing its adverse effects on the activation of anti-Glucose transporter (GLUT-4).

Similarly, IL-17, another pro-inflammatory cytokine related to the immune profile, known as IL-17 producing T cells (Th17), is also significantly reduced after RYGB surgery [58]. Studies have reported that obese individuals, especially those with DM2, have high systemic levels of Th17 cytokines, especially IL-17.[59–61]. In addition to macrophages, the adipose tissue of obese individuals with insulin resistance shows a marked presence of T-cell populations that produce pro-inflammatory cytokines, such as IL-17, and this infiltration by T-cells increases with the degree of adiposity [58, 62]. Additionally, both IL-17 and IL-1β can promote insulin resistance in adipocytes and favor the development of DM2 in obese individuals [58, 63]. Owing to its prominent association with obesity and insulin resistance, IL-17 has also been the subject of studies in the context of RYGB surgery, with significant reduction postoperatively [64–66], further corroborating our findings.

To confirm that RYGB surgery has a remarkable ability to modulate the Th17 profile, we also evaluated the serum levels of IL-23, which is a promoter molecule in the Th17 profile [67]. Serum levels of IL-23 significantly reduced after surgery, which corroborates with other studies that found a decrease in IL-23 levels after bariatric surgery in patients with insulin resistance, particularly in the context of RYGB surgery [64, 67, 68]. We also found a positive correlation between the serum levels of IL-17 and IL-23 both before and after surgery in the BSG group, which reiterates the reports in literature regarding the production and concomitant action of these molecules. However, similar findings were not observed in the CG, which suggests that under certain unclear conditions, the development and presence of MS can deregulate this profile.

Increased levels of IL-1β are also related to insulin resistance and obesity [58, 69] and according to Maddur et al. IL-1β may act as the main regulator of the Th17 profile expansion in humans [70]. Contrary to our findings, recent studies that evaluated the modulation of IL-1β levels in the context of RYGB surgery showed no change in the levels of this pro-inflammatory cytokines after surgery [69, 71, 72]. Thus, our findings highlight that obese women who manifest MS may benefit from RYGB bariatric surgery, with a plausible reduction in insulin resistance.

IL-6 has pro-inflammatory properties and a significant positive correlation with abdominal adiposity. [73] It also plays an important role in the pathogenesis of atherosclerosis, insulin resistance, and DM2 [74, 75]. Therefore, lower levels of IL-6 are associated with a decrease in several metabolic parameters, such as fasting blood glucose, TG, and glycosylated hemoglobin, an increase in HDL, and consequently, an improvement in cardiovascular risk [72]. Although it has been reported that IL-6 levels did not change at 6 months after surgery [71] as in our study, some studies have however, reported significant postoperative reductions of this cytokine after RYGB surgery [44, 71, 76]. This may be related to weight loss in these patients since the significant decrease in IL-6 levels after bariatric surgery was associated with a reduction of more than 50% in BMI [55]. These data corroborate with our observations of a significant reduction in IL-6 levels in the BSG group post operatively compared to only their preoperative levels, but not the levels in the CG, since the decrease in BMI did not reach 50% after the surgery.

Finally, it is important to mention that the BSG group showed significant positive correlations between certain cytokines after RYGB, which was not observed preoperatively. This finding shows that the beneficial metabolic changes resulting from the surgery might have promoted the restoration of inflammatory responses previously altered by obesity and MS. Corroborating this suggestion, the CG showed no correlation between the evaluated cytokines, both in the first and second evaluations, suggesting that the development of obesity and the manifestation of MS results in an individual pattern of systemic inflammation; therefore, correlations between these molecules may be difficult to determine.

### Study limitations

In order to generalize the clinical findings of this study to the general population, we must consider a limitation in the selection process of the patients involved. At the beginning of the study, in the enrollment phase, patients of both genders were invited to participate in this clinical trial. However, the number of male patients was much smaller when compared to the number of female patients, which could compromise the statistical analysis. In this sense, we chose to analyze data only from women with severe obesity undergoing RYBS-type bariatric surgery. Given this situation, we hope in the near future to present new clinical data from male patients with severe obesity undergoing RYBS and to compare the outcomes with female patients. Another limitation of the study was the fact that the study was blind only to the statistician who processed the data. Due to the intervention being a major surgery, the researchers could not be blinded to the results, as from an ethical point of view it would not be permissible to use a Sham group.

In contrast to this limitation, it can be highlighted as a strong point that a controlled, randomized study was conducted, where the number of patients involved in the final analysis of the data exceeded the sample calculation performed previously.

## Conclusions

According to our results, the reduction of anthropometric measurements, such as body weight and BMI, induced by bariatric surgery with RYGB, significantly improves not only the plasma biochemical parameters (lipid profile and glycemia), but also the systemic inflammatory status of severely obese patients with MS.

## Data Availability

The datasets generated or analyzed during the current study are available from the corresponding author on reasonable request.

## Abbreviations

MS: Metabolic syndrome
DM2: Type 2 diabetes mellitus
TG: Triglycerides
IL-6: Interleukin-6
TNF-α: Tumor necrosis factor-alpha
RYGB: Roux-en-Y gastric bypass
BMI: Body mass index
CG: Control group
BSG: Bariatric surgery group
TC: Total cholesterol
HDL: High-density lipoprotein
LDL: Low-density lipoprotein
